# Early Visual Cortices Reveal Interrelated Item and Category Representations in Aging

**DOI:** 10.1523/ENEURO.0337-23.2023

**Published:** 2024-03-12

**Authors:** Claire Pauley, Anna Karlsson, Myriam C. Sander

**Affiliations:** ^1^Center for Lifespan Psychology, Max Planck Institute for Human Development, Berlin 14195, Germany; ^2^Faculty of Life Sciences, Humboldt-Universität zu Berlin, Berlin 10115, Germany

**Keywords:** aging, episodic memory, fMRI, neural dedifferentiation, pattern similarity analyses

## Abstract

Neural dedifferentiation, the finding that neural representations tend to be less distinct in older adults compared with younger adults, has been associated with age-related declines in memory performance. Most studies assessing the relation between memory and neural dedifferentiation have evaluated how age impacts the distinctiveness of neural representations for different visual categories (e.g., scenes and objects). However, how age impacts the quality of neural representations at the level of individual items is still an open question. Here, we present data from an age-comparative fMRI study that aimed to understand how the distinctiveness of neural representations for individual stimuli differs between younger and older adults and relates to memory outcomes. Pattern similarity searchlight analyses yielded indicators of neural dedifferentiation at the level of individual items as well as at the category level in posterior occipital cortices. We asked whether age differences in neural distinctiveness at each representational level were associated with inter- and/or intraindividual variability in memory performance. While age-related dedifferentiation at both the item and category level related to between-person differences in memory, neural distinctiveness at the category level also tracked within-person variability in memory performance. Concurrently, neural distinctiveness at the item level was strongly associated with neural distinctiveness at the category level both within and across participants, elucidating a potential representational mechanism linking item- and category-level distinctiveness. In sum, we provide evidence that age-related neural dedifferentiation co-exists across multiple representational levels and is related to memory performance.

## Significance Statement

Age-related memory decline has been associated with neural dedifferentiation, the finding that older adults have less distinctive neural representations than younger adults. This has been mostly shown for category information, while evidence for age differences in the specificity of item representations is meager. We used pattern similarity searchlight analyses to find indicators of neural dedifferentiation at both levels of representation (category and item) and linked distinctiveness to memory performance. Both item- and category-level dedifferentiation in the calcarine cortex were related to interindividual differences in memory performance, while category-level distinctiveness further tracked intraindividual variability. Crucially, neural distinctiveness was strongly tied between the item and category levels, indicating that intersecting representational properties of posterior occipital cortices reflect both individual exemplars and categories.

## Introduction

In older age, episodic memories tend to lose the amount of detail with which they can be recovered, rendering older adults more susceptible to memory errors and forgetting. According to prominent theories of cognitive aging, such a loss of mnemonic information may be linked to age-related changes in the quality of the underlying neural representations (Li and Lindenberger, 1999). Specifically, the distinctiveness of neural representations is reduced in older compared with younger adults, a phenomenon termed “age-related neural dedifferentiation” (for reviews, see Koen and Rugg, 2019; Sommer and Sander, 2022). Past studies have operationalized neural distinctiveness as the selectivity of univariate activation responses in ventral visual cortices to stimuli belonging to different categories (i.e., category-level distinctiveness; Park et al., 2004). However, it is currently unknown how aging impacts the distinctiveness of representations for individual events (i.e., item-level distinctiveness).

Multivariate analysis techniques, such as representational similarity analysis (RSA), can be leveraged to investigate age differences in neural distinctiveness not only at the category level (Carp et al., 2011; Zheng et al., 2018; Bowman et al., 2019; Koen et al., 2019; Srokova et al., 2020; Hill et al., 2021; Dennis and Koen, 2022; Koen, 2022; Pauley et al., 2023), but also at the level of individual stimuli, though this has rarely been done. One exception comes from Trelle et al. (2019) who showed that the representational stability of individual object and scene stimuli across multiple presentations was reduced in older compared with younger adults in the ventral temporal cortex (see also, Koen, 2022; for absent age effects, see Zheng et al., 2018). Thus, while age-related neural dedifferentiation presumably manifests across both category- and item-representational levels, evidence of age differences in item-level distinctiveness is meager.

Consequently, it is unknown whether age impacts category- and item-level distinctiveness similarly or differentially (Sommer and Sander, 2022) and how the two interact in service of memory. Understanding how category- and item-level distinctiveness are related is crucial to elucidating the mechanism(s) driving representational distinctiveness and, in turn, age-related neural dedifferentiation. If category- and item-level distinctiveness are strongly interrelated, and thus similarly affected by age, it is likely that highly overlapping representational properties drive distinctiveness. For example, it has been shown that categorical distinctiveness emerges due to commonalities across many exemplars of a given category (Long et al., 2018), suggesting a tight link between the two levels of distinctiveness. On the other hand, if category- and item-level distinctiveness have dissociable underlying representational properties, we would expect them to not be related and accompanied by differential age-related effects. A recent study (Koen, 2022) reported that representational similarity was strongly correlated across items and categories, but that age differences in similarity between items and categories were unrelated, concluding that age-related decline across these two levels of representation stem from differential mechanisms (see also, Srokova et al., 2023). However, neither item nor category similarity was associated with memory performance, thus, their methods may not have sufficiently targeted memory-related representations.

Importantly, theories of cognitive aging propose that less distinct neural representations at the item level contribute to mnemonic impairments in older adults (Li et al., 2001). In line with this proposal, previous studies have shown that individuals with less distinctive representations tend to perform worse on episodic memory tasks (e.g., Zheng et al., 2018) regardless of age (Koen et al., 2019). However, while the relationship between interindividual differences in neural distinctiveness of categorical representations and memory performance has been substantiated in both younger and older adults, whether across-person variability of item-level distinctiveness predicts memory outcomes has not been evaluated so far. Furthermore, several studies have related trial-by-trial variability in neural distinctiveness within individuals to subsequent memory outcomes, but these were restricted to younger adults (Kuhl et al., 2012; Xue et al., 2010, 2013; Lu et al., 2015; Hasinski and Sederberg, 2016; Xue, 2018; Zheng et al., 2018). Only one study has suggested this association in older adults (Zheng et al., 2018). Altogether, despite its distinguished role in cognitive aging research (Koen and Rugg, 2019), it is currently not understood how age-related neural dedifferentiation manifests across different representational levels and relates to memory outcomes both at the individual level as well as on the trial level.

We analyzed fMRI data from younger and older adults while they studied objects across multiple presentations and completed a subsequent memory task. We first used exploratory pattern similarity searchlight analyses to identify brain regions exhibiting age differences in category- and item-level distinctiveness. Congruent with the neural dedifferentiation hypothesis, we expected reduced distinctiveness across both levels in older compared with younger adults. We further examined the relationship between age differences in distinctiveness and memory performance both within and across individuals, predicting that more distinct representations would be associated with better memory outcomes. Finally, we assessed the relationship between age deficits in category- and item-level distinctiveness. We hypothesized that the two levels of distinctiveness would be interconnected, in line with the idea that distinctiveness for categories incidentally manifests due to perceptually similar features of individual exemplars from a particular category.

## Materials and Methods

Behavioral and electroencephalographic (EEG) data from this project were previously reported in two papers (Karlsson et al., 2022; Karlsson and Sander, 2023). Behavioral data relevant to the present study are re-analyzed here.

### Participants

Data were collected from a total of 141 healthy, right-handed adults. Participants were recruited within two age groups: younger adults (20–31 years, *N* = 67) and older adults (64–76 years, *N* = 74). Thirteen participants were excluded due to technical issues (eight younger adults and five older adults), eight were excluded due to too much motion in the scanner (see fMRI data acquisition and preprocessing, for motion criterion; three younger adults and five older adults), one older adult was excluded for having below-chance memory performance (see Behavioral analyses, for behavioral threshold), seven participants dropped out (two younger adults and five older adults), and 12 participants were excluded due to missing fMRI data (six younger adults and six older adults). The final sample consisted of 48 younger adults (*M*_age_ = 24.8 years, *SD*_age_ = 3.0 years, 28 females, 20 males) and 52 older adults (*M*_age_ = 69.6 years, *SD*_age_ = 3.7 years, 27 females, 25 males). Participants were screened via telephone for mental and physical illness, metal implants, and current medications. The study was approved by the Ethics Committee of the German Society for Psychological Research (DGPs) and written informed consent was obtained from each participant at the time of the study. Finally, all older adults were screened using the Mini-Mental State Examination (Folstein et al., 1975) and all exceeded the threshold of 24 points indicating normal cognition.

### Stimuli

The stimulus pool was comprised of 498 colored images (400 × 400 pixels) of everyday objects (e.g., ball, teapot; Brady et al., 2008) and 210 colored images (1,280 × 960 pixels) of outdoor scenes (e.g., forest, beach) retrieved from the internet (open source).

### Experimental design

**Figure 1.**
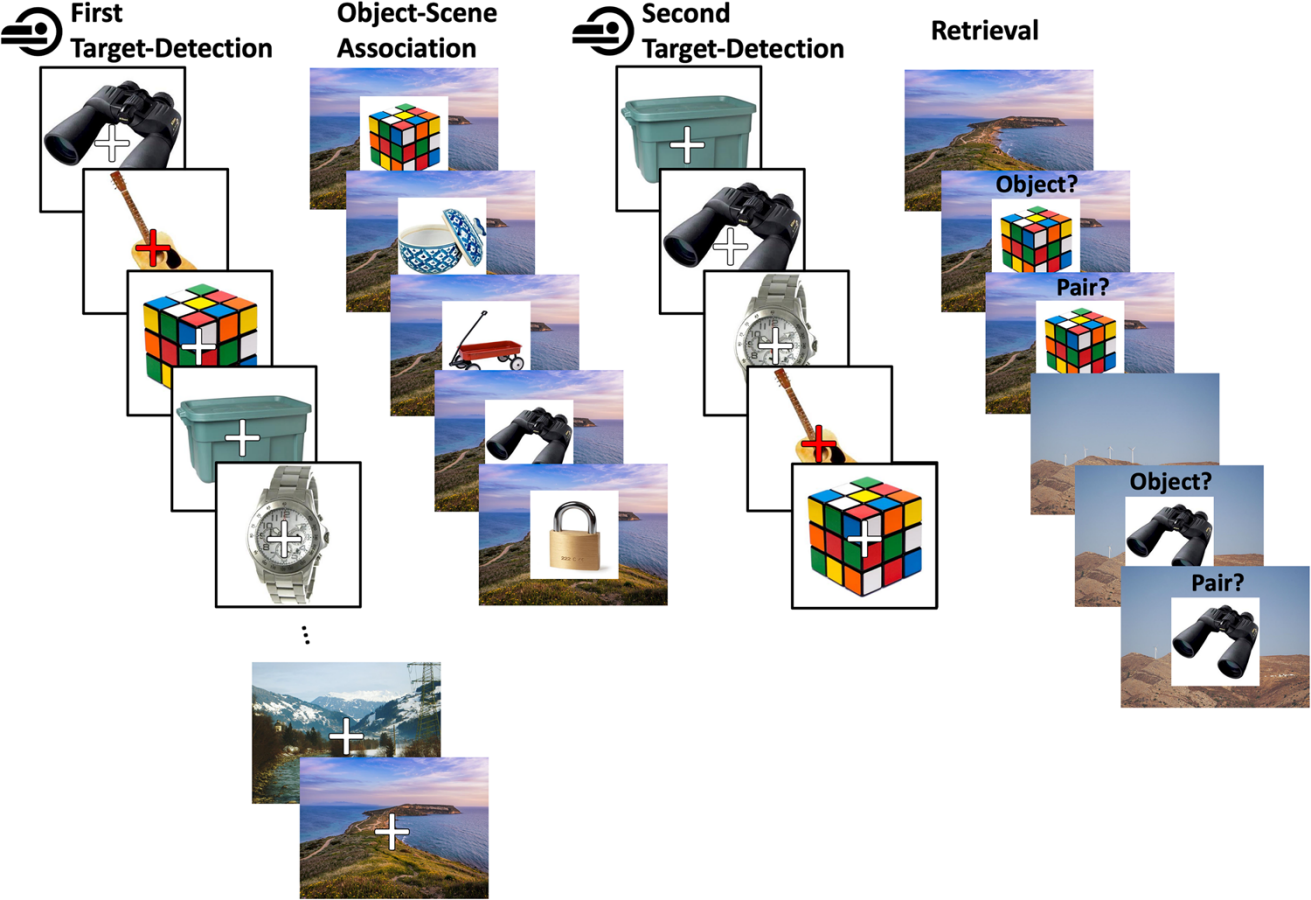
Experimental design consisting of four phases: first target-detection, object–scene association, second target-detection, and retrieval. The first and second target-detection phases were completed in the fMRI scanner. In the first target-detection phase, participants were shown a series of objects followed by a series of scenes while completing a target-detection task. In the object–scene association phase, objects were superimposed on scenes and participants were instructed to imagine using the object in the place depicted. During the second target-detection phase, objects were presented one-by-one again (in pseudorandomized order). Finally, during retrieval, objects (either old or new) were superimposed on scenes (either old or new) and participants were asked, first, whether the object was old or new, and then, whether the object–scene pair was old or new.

The experiment consisted of four parts: a first target-detection phase, an object–scene association phase, a second target-detection phase, and a retrieval phase (Fig. 1). All phases were completed in a single day with short breaks between the sessions, including a 40 min lunch break after the object–scene association phase. During the first target-detection phase, participants completed a target-detection task in the MRI scanner while viewing object and scene stimuli. Participants were then removed from the scanner to complete an object–scene association task, during which EEG and eye movement data were collected. In the second target-detection phase, participants again completed a target-detection task in the MRI scanner while viewing the object stimuli. Finally, participants performed an old/new recognition memory task in which objects were presented with scenes and participants were asked whether the object was old or new and whether the specific object–scene pair was old or new. The present study focuses on the fMRI data from the first and second target-detection phases and assesses relationships between neural measures of cortical distinctiveness and outcomes on the recognition test. Effects of the object–scene association task on neural representations in the hippocampus will be reported elsewhere. As elaborated in Searchlight similarity metrics section, control analyses were conducted to rule out that the association task had effects on cortical representations of objects or scenes.

The first target-detection phase consisted of four runs: three object runs and one scene run. Each trial started with a white fixation cross in the middle of the screen for 2,307 ms, followed by the stimulus presentation for another 2,307 ms, and finally another fixation cross for 2,307 ms. Throughout all runs, participants were asked to complete a target-detection task in which they pressed a button when the fixation cross superimposed on the stimulus turned red. For the object runs, a total of 320 objects were presented in pseudorandom order with 106 objects shown in the first run and 107 in both the second and third runs. Twenty objects served as target images in the target-detection task and were excluded from all analyses. Of the remaining objects, 250 were later paired with scenes in the object–scene association phase (see below) and 50 served as so-called “baseline” objects that were not included in any further task. In the scene run, 60 scenes were shown in total, 10 of which were target images and the remaining 50 were included in the object–scene association task.

During the object–scene association phase, 250 objects were randomly paired with the 50 old scenes and presented together in an object–scene association task (described in detail in Karlsson et al., 2022).

In the second target-detection phase, the same 320 objects from the first target-detection phase were presented in pseudorandomized order, split into 3 runs, while participants completed the same target-detection task. The scenes were not presented in the second target-detection phase.

Finally, in the retrieval phase, the 250 objects from the object–scene association phases were presented intermixed with 150 new objects. Both old and new objects were presented either on an old or on a new scene. Among the old objects, some of them were presented on an old matching scene (*n* = 100) from study, an old mismatching scene (*n* = 100), or on a new scene (*n* = 50). For the purpose of the present study, analyses were collapsed across these context conditions, focusing on item and pair memory regardless of context condition at retrieval. Each trial began with a centered fixation cross for 0.5 s, followed by a scene for 1 s, and then an object was superimposed on the scene. Participants were first asked whether the object was old or new, to which they had 3 s (max.) to respond, and subsequently asked whether the specific object–scene pair was old or new, to which they had 4 s (max.) to respond. Responses were recorded using a response box and the mapping of button to response was counterbalanced across participants.

### fMRI data acquisition and preprocessing

Brain imaging was acquired with a Siemens Magnetom TrioTim 3T MRI scanner with a 32-channel head-coil. MRI data collection took place across two sessions on the same day (first and second target-detection). In the first session, anatomical, functional, and hippocampal structural scans (structural scans were not analyzed here) were collected and only functional scans were collected in the second session. Two anatomical T1-weighted magnetization prepared rapid acquisition gradient echo (MPRAGE) pulse sequence images were acquired: one with distortion correction and one without (192 slices; voxel size = 1 × 1 × 1 mm^3^; TR = 2,500 ms; TE = 4.77 ms; flip angle = 7°; FoV = 256 mm). Functional images were acquired using a multiband gradient echo planar imaging (EPI) sequence (multiband acceleration factor = 4; voxel size: 1.5 × 1.5 × 1.5 mm^3^; TR = 2,307 ms; TE = 32 ms; flip angle = 71°; FoV = 192 mm). The image acquisition was positioned to avoid signal loss in the medial temporal lobe, but consequently resulted in minor signal loss in posterior occipital cortices. Ten additional functional volumes were collected in both scanning sessions with opposite phase-encoding directions (5 volumes in each direction) for subsequent susceptibility distortion correction. Experimental stimuli, which participants viewed via a mirror mounted on the head-coil, were projected using the Psychtoolbox (Psychophysics toolbox) for MATLAB (Mathworks Inc.).

MRI data were organized according to the Brain Imaging Data Structure (BIDS) specification (Gorgolewski et al., 2016) and preprocessed using *fMRIPrep* (version 1.3.2; Esteban et al., 2019) with the default settings. The T1w image was corrected for intensity nonuniformity and skull-stripped. Functional images were motion-corrected, slice-time corrected, and co-registered to the T1w reference image. Transformation matrices between T1w space and the *ICBM 152 Nonlinear Asymmetrical template version 2009c* were generated. Finally, functional images were resampled to 2 mm isotropic voxels in order to enhance the signal-to-noise ratio (Dimsdale-Zucker and Ranganath, 2018).

Visual inspection of the preprocessed fMRI data revealed eight participants with strong effects of motion, defined as having at least three functional runs with six or more framewise displacement measures greater than voxel size (1.5 mm) corresponding with evident artifacts in carpet plots (Power, 2017). These individuals were excluded from further analyses (see also Participants).

### Behavioral analyses

Two measures of memory were assessed: item (memory for objects) and pair (memory for object–scene pairs). Memory performance was calculated as the proportion of correct item/pair responses (collapsing across hits and correct rejections). For each participant, the proportion of correct item responses was compared to the chance level of 0.31 (derived from the multiplication of the response probability by the proportion of trials with an old object, i.e., 0.5 × 250/400 = 0.31). Participants with item memory performance below chance were excluded from further analyses (see also Participants; Karlsson et al., 2022). A two-way mixed factorial ANOVA was used to assess memory performance for age differences and differences between memory measure (item vs pair). Significant interactions were followed up by pairwise comparisons and reported *p*-values were Bonferroni-corrected. We additionally performed an independent-samples *t*-test to assess age differences in response bias for item memory judgments defined as FAR/(1 − (HR − FAR)), where FAR is the false alarm rate and HR is the hit rate (Snodgrass and Corwin, 1988).

### Pattern similarity searchlight analyses

First, using Statistical Parametric Mapping software (SPM 12; Wellcome Trust Centre for Neuroimaging), a general linear model was performed for each trial in both the first and second target-detection phases consisting of one trial-specific regressor, one regressor for all other trials in the same run, six motion regressors, and one regressor modeling the first three “dummy” volumes of each run. Trial regressors were modeled as event-related stick functions corresponding with the onset of each stimulus convolved with a canonical hemodynamic response function. Due to the large amount of signal loss in occipital, medial temporal, and medial prefrontal cortices resulting in many voxels not passing the strict noise threshold set in the SPM default settings, the implicit masking threshold was set to 0.4 (for similar methodology, see Maass et al., 2019). Following this threshold adjustment, we still find signal loss in frontal (13% loss), temporal (6% loss), occipital (5% loss), and parietal (2% loss) cortices. Pattern similarity analyses were conducted on the resulting *β* weights for each trial in T1w space. Pattern similarity was only assessed between trials from different runs to control for time-dependent correlations in the hemodynamic responses (Dimsdale-Zucker and Ranganath, 2018) and was measured as Fisher *z*-transformed Pearson correlations. Searchlight similarity analyses were conducted using modified scripts from the MATLAB toolbox for representational similarity analysis (Nili et al., 2014) and with 4-mm-radius spherical searchlights resulting in whole-brain maps of mean similarity values for each similarity metric (see below for descriptions of similarity metrics). Brain maps for each searchlight similarity metric were normalized to MNI space using *antsApplyTransforms* and the transformation matrix generated by *fMRIPrep*. Only voxels in which searchlight similarity metrics were available in all participants were considered in subsequent analyses.

Thresholding for all contrasts was performed using nonparametric, cluster-based, random permutation analyses adapted from the FieldTrip toolbox (Oostenveld et al., 2011) within each age group. First, dependent-samples *t*-tests were conducted within each voxel. Adjacent voxels with significance values lower than a threshold of *p* < 0.005 were clustered together (*p* < 0.01 for the control analyses). The sum of all *t* statistics of the voxels included in each cluster was taken as the cluster test statistic. The Monte Carlo method was used to determine whether a particular cluster was significant by comparing the cluster test statistic to a reference distribution of *t* statistics across 1,000 permutations. Each *t* statistic in the reference distribution was created by randomly shuffling the two conditions and recalculating the cluster test statistic. Clusters were considered significant under a threshold of *p* < 0.05 and if they contained at least 10 voxels.

### Searchlight similarity metrics

#### Control analysis

The present experimental paradigm included an object–scene association phase in which objects were associated with scenes with the aim to investigate how object–scene associations are represented in the hippocampus (to be reported elsewhere). To control for potential effects of the object–scene association phase on the cortical neural distinctiveness measures reported here, we ran several control similarity searchlight analyses. First, we looked at whether the across-voxel correlation between objects paired to the same scene (i.e., within-scene similarity) were more similar in the second target-detection phase compared with the first target-detection phase, which would indicate that the scene was represented in the object patterns. To control for scanning session-related artifacts, we subtracted the similarity of objects paired to different scenes (i.e., between-scene similarity) from within-scene similarity within each scanning session (e.g., second target-detection within-scene similarity – second target-detection between-scene similarity) before contrasting first and second target-detection. Second, we reran the comparison of objects associated with the same scene compared to objects associated with different scenes restricted to objects with correct subsequent pair memory response (hit or correct rejection) in the recognition task with the assumption that any learning associated changes were more likely to occur for correct rather than incorrect pair memory response. Third, we reran the same comparison, but increased the power by splitting the scenes into strong-memory scenes (scenes in which three to five of the object–scene pairs were correctly remembered) and weak-memory scenes (scenes in which zero to two of the object–scene pairs were correctly remembered) and considering all objects belonging to the strong-memory scenes. None of these control analyses indicated any effect of the learning task on the cortical representation of the objects (*p*s > 0.20).

#### Main analysis

**Figure 2.**
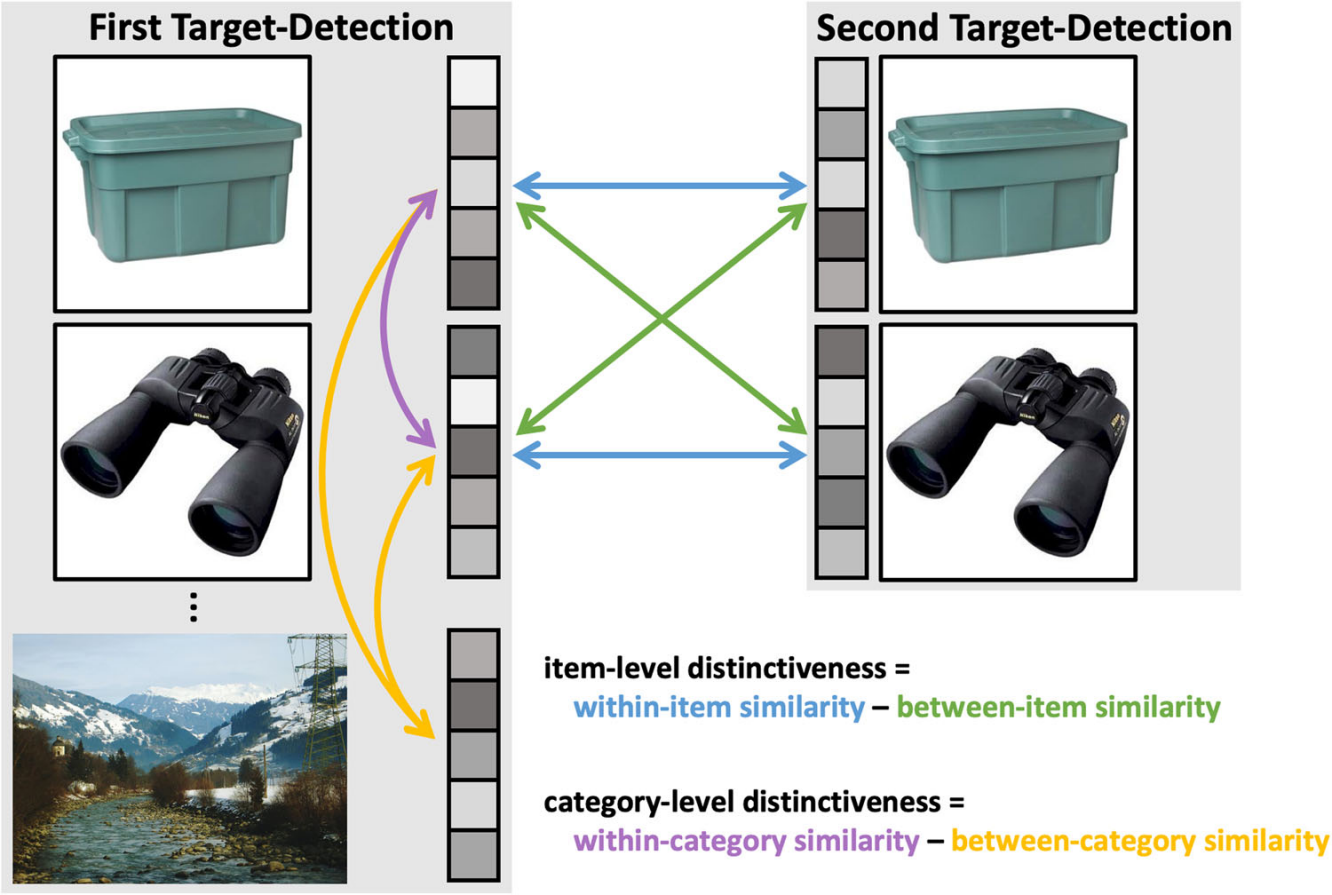
Illustration of item- and category-level distinctiveness metrics. Item-level distinctiveness was measured as the difference between within-item similarity (i.e., the across-voxel correlation of the same object from the first and second target-detection phases; shown in blue) and between-item similarity (i.e., the similarity of one object from first target-detection to all other objects from second target-detection; shown in green). Category-level distinctiveness was calculated as the difference between within-category similarity (i.e., the similarity between an object and all other objects from first target-detection; displayed in purple) and between-category similarity (i.e., the similarity between objects and scenes; displayed in yellow).

We first ran searchlight similarity analyses looking for regions demonstrating high neural distinctiveness. We were interested in assessing two representational levels of distinctiveness: item and category (for visualization, Fig. 2). Since only objects (not scenes) were shown in the second target-detection phase, we only measured distinctiveness for objects. To assess item-level distinctiveness, we contrasted within-item similarity and between-item similarity. Within-item similarity was defined as the across-voxel correlation of an object's activity pattern during first target-detection to the same object's activity pattern during the second target-detection phase. Between-item similarity was defined as the mean similarity of the activity patterns from all objects during the first target-detection phase to all objects during the second target-detection phase, excluding the within-item similarity values. To assess category-level distinctiveness, we contrasted within-category similarity and between-category similarity. Since scenes were only shown during the first target-detection phase, within-category and between-category similarity were derived only from the first target-detection phase. Within-category similarity was defined as the averaged across-voxel correlation of the activity pattern in response to a given object to the activity patterns in response to all other objects. Between-category similarity was defined as the mean similarity of the activity patterns from all objects to all scenes. The anatomical regions to which (at least some) voxels of the resulting clusters belong are listed in the results based on the automated anatomical labelling (AAL; Tzourio-Mazoyer et al., 2002) atlas.

### Assessing age differences in neural distinctiveness

Next, we were interested in searching the whole brain for regions demonstrating age differences in both item- and category-level distinctiveness using cluster permutation analyses. First, independent-samples *t* tests were conducted within each voxel comparing younger and older adults on each distinctiveness level. Adjacent voxels with significance values lower than a threshold of *p* < 0.005 were grouped into clusters and the sum of all *t* statistics of the voxels included in each cluster was defined as the cluster test statistic. The Monte Carlo method was used to determine whether a cluster was significant. In this case, the reference distribution was created by removing the younger and older adult labels and randomly assigning participants to each age group across 1,000 permutations. In order to run follow-up analyses relating age differences in neural distinctiveness to memory performance, clusters demonstrating age differences (three in total, two item level and one category level) were transformed back into T1w space and were used as masks to generate trial-wise and participant-wise measures of neural distinctiveness.

### Relating age differences in neural distinctiveness to memory performance

We first assessed how interindividual differences in neural distinctiveness relate to memory performance. We implemented partial least squares correlation (PLSC) analyses in order to understand the common impact of age differences in neural distinctiveness at both the item- and category-levels on memory performance as well as to delineate the weights of the individual contributions of neural distinctiveness at each level (Krishnan et al., 2011). We used PLSC because it offers the unique advantage of relating multiple behavioral and multiple neural measures in a single model, rather than several correlational models and also without introducing multicollinearity artifacts (as with multiple regression; Gregorich et al., 2021). First, a between-person correlation matrix was calculated between a *n *× 2 matrix containing item and pair memory performance (*Pr*) and a *n *× 3 matrix of neural distinctiveness from the 3 clusters showing age differences. This correlation matrix then underwent singular value decomposition, producing a single estimate latent variable (LV) that optimally represents the association between neural distinctiveness and memory performance. The significance of the LV was tested using 10,000 permutation tests of the singular value corresponding to the LV. Robustness estimates were measured using a bootstrapping procedure across 10,000 resamples of the data. Bootstrap ratios (BSRs; normalized robustness estimates) were then calculated by dividing the neural distinctiveness weights from the singular value decomposition by the standard errors of their robustness estimates. Similar to *z* values, BSRs are considered reliably robust with values above or below ±1.96, which we used to delineate which level(s) of neural distinctiveness were reliably associated with memory performance.

In order to understand whether age differences in neural distinctiveness related to *intra*individual differences in memory performance, we performed three generalized linear mixed effects models (one per previously identified cluster showing age differences) in which memory outcomes (item hit or miss) were predicted by neural distinctiveness. For the two item-level clusters, trial-wise item-level distinctiveness (operationalized as the difference between the similarity of an object to itself across first and second target-detection and the mean similarity of the same object to all other objects) was used as an independent variable, and for the category-level cluster, category-level distinctiveness (operationalized as the difference between the mean similarity of an object from the first target-detection phase to all other objects and the mean similarity of the same object to all scenes) was assessed as an independent variable. The interaction between age and neural distinctiveness was included in the models. The models were analyzed using the *R* function *glmer* from the lme4 package with the following formula: Memory ∼ Age × Distinctiveness + (1 + Distinctiveness | Subject).

Our trial-wise analyses yielded no intraindividual relationships between neural distinctiveness and memory performance (see section “Neural distinctiveness related across representational levels”). We suspected this might be due to the fact that this analysis was based on brain regions demonstrating age differences in neural distinctiveness, thus highlighting the interindividual differences in distinctiveness. Therefore, we ran an additional exploratory whole-brain searchlight analysis in order to investigate whether any regions demonstrated greater neural distinctiveness (at either the category or item level) for objects later remembered versus forgotten. We split each distinctiveness searchlight measure based on subsequent item-memory outcome. For example, for item-level distinctiveness, we averaged within-item and between-item similarity first across trials that were subsequently remembered and then across trials that were subsequently forgotten. Then, distinctiveness (e.g., for the item level, the difference between within-item and between-item similarity) was calculated separately for remembered and forgotten objects within each participant and permutation analyses were performed to test for differences based on subsequent memory as well as age differences in this effect (see Pattern similarity searchlight analyses and Assessing age differences in neural distinctiveness for analysis details).

### Relating age differences in neural distinctiveness across representational levels

We examined whether age differences in neural distinctiveness were related across the item and category level. As reported in the results (see Results, Inter- and intraindividual variability in distinctiveness relates to memory performance), one of the item-level clusters demonstrated age-related neural dedifferentiation, as did the category-level cluster. Thus, we related neural distinctiveness measures extracted from these two clusters in order to assess the relationship between item- and category-level distinctiveness. First, to assess the relationship across participants, we performed a linear regression in which we tested whether item-level distinctiveness could be predicted from category-level distinctiveness. The model was analyzed using the *R* function *lm* and the following formula: Item ∼ Age × Category. Finally, we assessed whether trial-by-trial neural distinctiveness across representational levels was related using a linear mixed effects model with the following formula: Item ∼ Age × Category + (1 + Category | ID).

## Results

### Behavioral results

**Figure 3.**
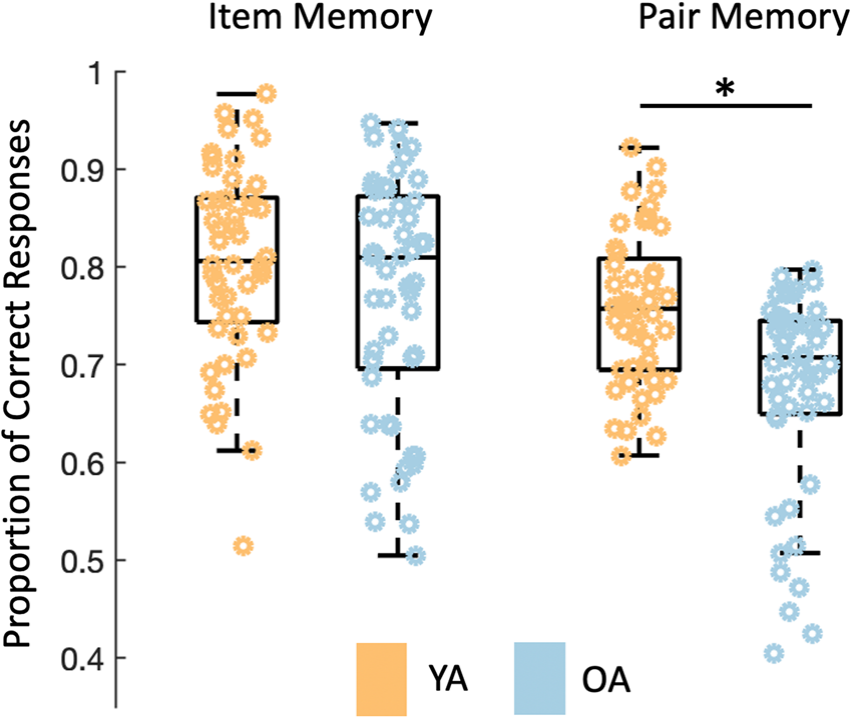
Proportion of correct responses with respect to item memory (left) and pair memory (right). No age differences were found for item memory between younger (YA; orange) and older (OA; blue) adults, but younger adults had a higher proportion of correct pair-memory responses than older adults. Box plots represent the interquartile range (first and third quantile with a median center line), with dots representing individual participants.

Age differences in memory performance were assessed using a two-way mixed factorial ANOVA with age (younger/older) as the between-subjects factor and memory level (item/pair) as the within-subjects factor. Results revealed an age-by-memory level interaction (Fig. 3; *F*_(1,98)_ = 5.08, *p* = 0.027, partial-*η*^2^ = 0.02). Follow-up comparisons demonstrated no age differences in item-memory performance (*t*_(98)_ = 1.48, *p* = 0.14, *d* = 0.29), but age differences in pair-memory performance (*t*_(98)_ = 4.34, *p* < 0.001, *d* = 0.86), with younger adults (*M *± *SD* = 0.76 ± 0.08) giving more correct responses than older adults (*M *± *SD* = 0.67 ± 0.12). No age differences in response bias for items were observed (*t*_(98)_ = 1.20, *p* = 0.23, *d* = 0.24).

### Pattern similarity searchlights reveal item- and category-level distinctiveness in occipital and ventral visual regions

**Figure 4.**
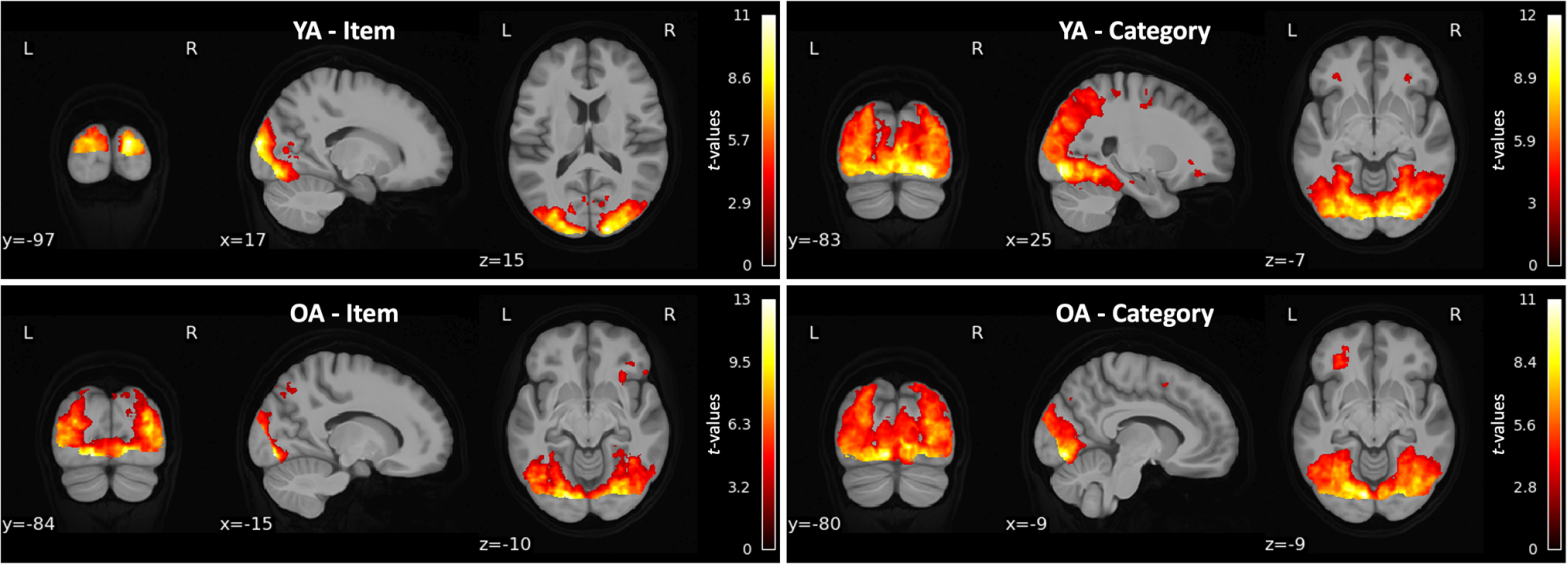
Regions demonstrating neural distinctiveness at the item (left column) and category (right column) level in younger (YA; top row) and older (OA; bottom row) adults. Peak loci of each cluster are reported in the Extended Data (Figure 4-1).

Using a whole-brain pattern similarity searchlight approach, we looked for regions demonstrating both item-level (greater within-item similarity than between-item similarity) and category-level (greater within-category than between-category) distinctiveness within each age group (Fig. 4). In both younger and older adults, a cluster of item-level distinctiveness was found in occipital cortices (*p* < 0.001), including in the bilateral middle occipital and fusiform gyri. In older adults, item-level distinctiveness was found in three additional clusters in inferior frontal cortices (*p*s = 0.002), including the inferior orbital, opercular, and triangular frontal gyri. Category-level distinctiveness was revealed in bilateral occipital and ventral visual cortices in both age groups (*p* < 0.001), including in the middle occipital and fusiform gyri. Six additional clusters of category-level distinctiveness were found in younger adults in temporal and frontal cortices, including the superior and middle temporal gyri as well as inferior orbital, triangular, and opercular frontal gyri (*p*s < 0.05). Three additional clusters were identified in older adults in the bilateral inferior triangular frontal, inferior orbitofrontal, and middle frontal gyri (*p*s < 0.005). Peak loci of each cluster are reported in the Extended Data.

10.1523/ENEURO.0337-23.2023.f4-1 Figure 4-1 Clusters identified by searchlight similarity analyses revealing high item- and category-level distinctiveness in younger and older adults. Download Figure 4-1, DOCX file.

### Age differences in item- and category-level distinctiveness

**Figure 5.**
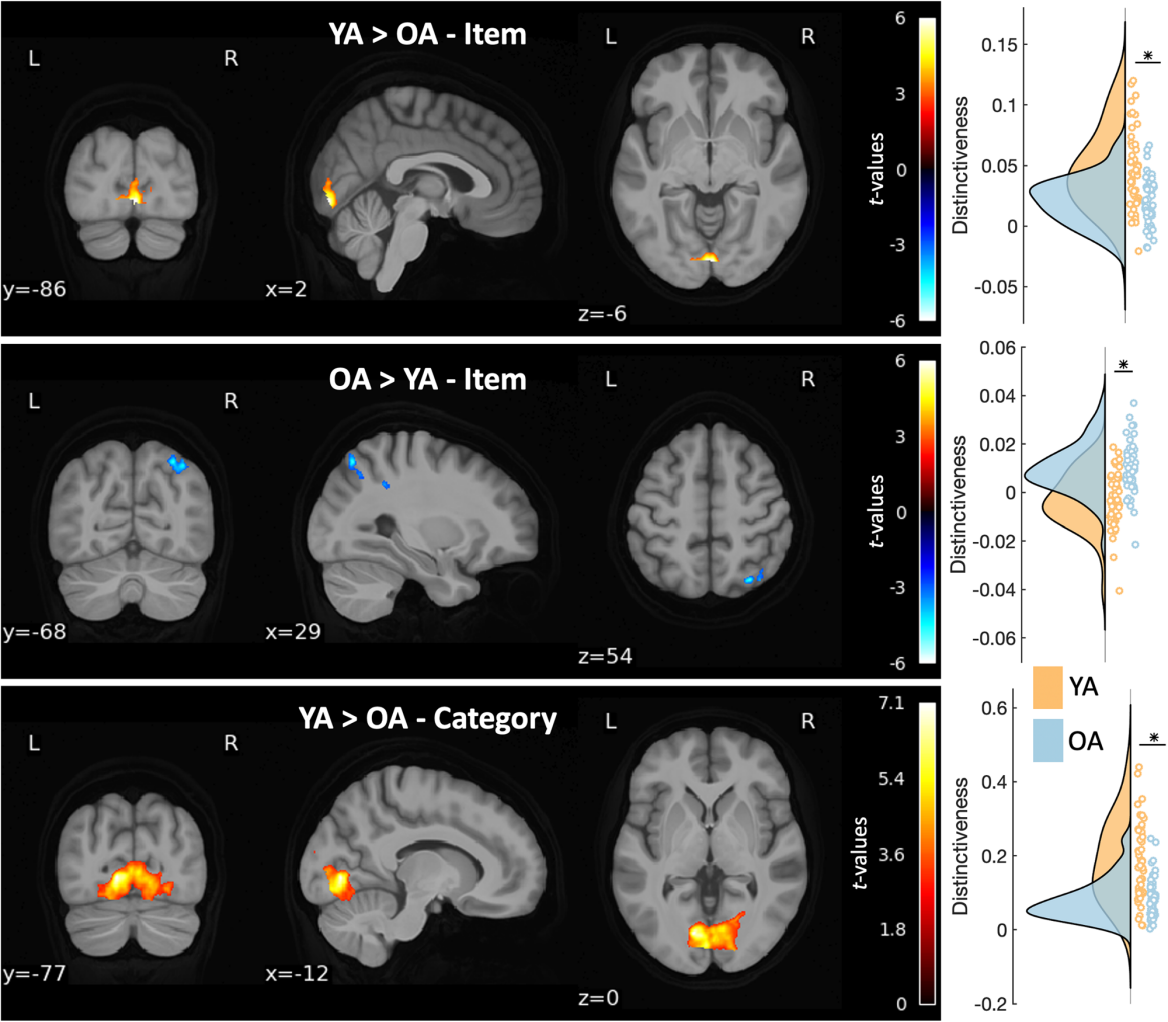
Regions demonstrating age differences in neural distinctiveness. At the item level, younger adults (YA; orange) had higher neural distinctiveness than older adults (OA; blue) in occipital cortices (top row), but lower distinctiveness in parietal cortices (middle row). At the category level, YA also showed higher neural distinctiveness than OA in occipital cortices (bottom row). Half-violin plots illustrate the sample density of distinctiveness within each region. Peak loci of each cluster are reported in the Extended Data (Figure 5-1).

Whole-brain pattern similarity searchlight analyses revealed two clusters of age differences in item-level distinctiveness: one in which younger adults demonstrated greater distinctiveness than older adults in occipital cortices (*p* = 0.02), including the calcarine cortex and lingual gyrus, and one in which older adults had more distinctive item-level representations than younger adults in posterior parietal cortices (*p* = 0.005), including the angular, superior parietal, and inferior parietal gyri (Fig. 5). On the category level, younger adults had greater distinctiveness than older adults in occipital cortices (*p* < 0.001), including the calcarine cortex and lingual gyrus.

10.1523/ENEURO.0337-23.2023.f5-1 Figure 5-1 Clusters revealed by searchlight similarity analyses demonstrating age differences in item- and category-level distinctiveness. Download Figure 5-1, DOCX file.

### Inter- and intraindividual variability in distinctiveness relates to memory performance

**Figure 6.**
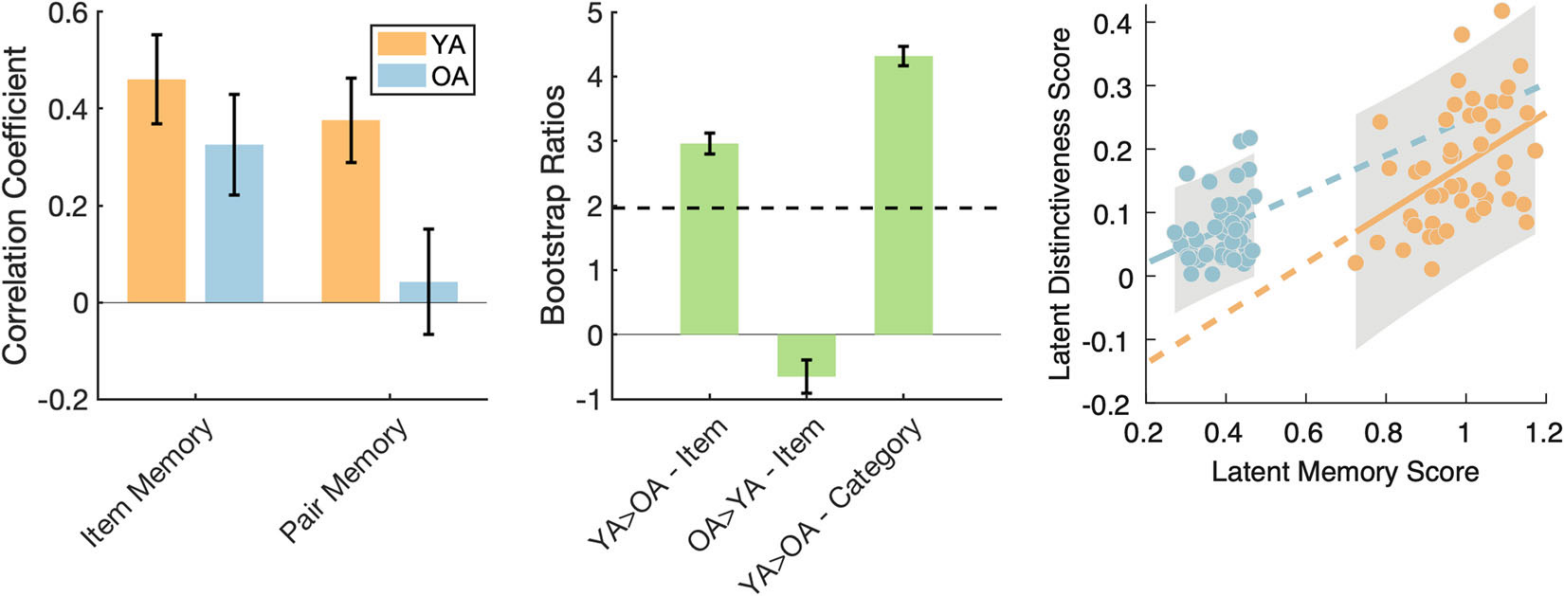
Relationship between neural distinctiveness and memory identified by PLSC. Correlation coefficients (left) of the significant latent relationship for item and pair memory in younger (YA; orange) and older (OA; blue) adults. Bootstrap ratios (middle) revealing age-related neural dedifferentiation at both the item and category level as the stable components with the robustness cutoff indicated by the dashed line. Higher neural distinctiveness was associated with better memory performance across individuals. Within-group least-squares lines are shown along with 95% confidence intervals (in gray). Solid lines indicate the within-group regression and dashed lines show the corresponding slopes projected along the whole x-axis to compare across groups.

In order to understand whether age differences in neural distinctiveness were associated with memory, we performed a PLSC analysis relating neural distinctiveness measure from each cluster identified in the searchlight analysis for age differences to both item and pair memory performance. This PLSC analysis revealed a single reliable latent variable (LV; *p* < 0.001) that optimally represents the relationship between neural distinctiveness and memory (Fig. 6; note that the relationship remains significant when standardizing all variables within age groups). Younger adults demonstrated modest latent correlations for both item and pair memory (*r* = 0.46 and 0.38, respectively), while older adults had weak latent correlations for item and pair memory (*r* = 0.33 and 0.04, respectively). Bootstrap ratios (BSRs) revealed the item- and category-level *de*differentiation clusters as the stable components of the LV explaining the largest amount of information common to memory performance and neural distinctiveness (BSRs = 2.19 and 4.32, respectively). The interaction of the directionality of the correlation coefficients and BSRs suggest a positive relationship, such that higher neural distinctiveness at both the item- and category-level (in the clusters showing dedifferentiation) is associated with better memory performance across individuals.

We were additionally interested in whether neural distinctiveness and memory were associated at the individual trial level. Using generalized linear mixed models (one model per cluster identified during the searchlight similarity analysis for age differences in distinctiveness), we predicted item memory outcomes (hit or miss) from trial-wise neural distinctiveness, along with the interaction between age and distinctiveness. No main effects or interactions reached significance (*p*s > 0.07).

**Figure 7.**
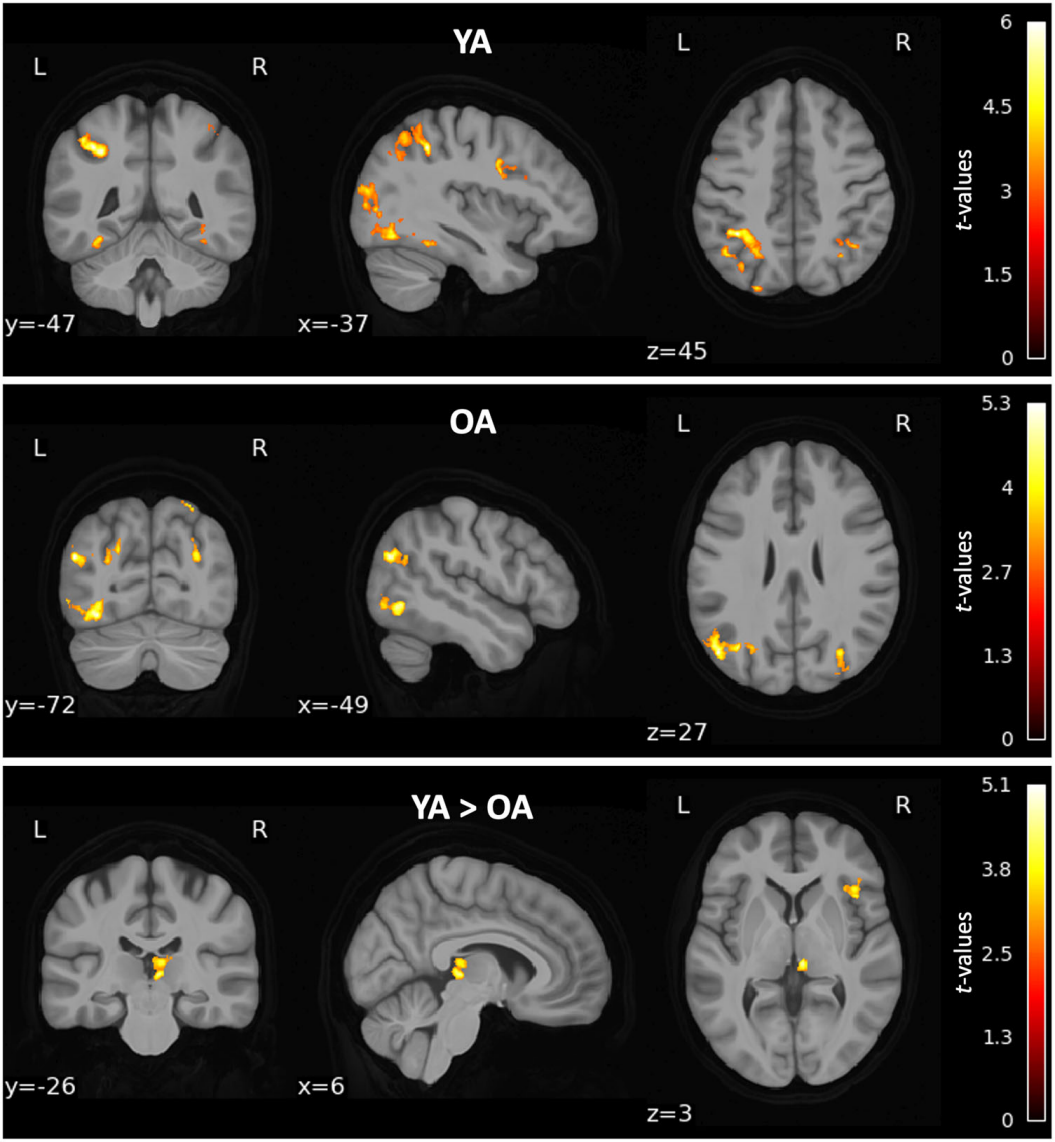
Regions demonstrating greater category-level distinctiveness for subsequently remembered objects than forgotten objects in younger adults (YA; top), older adults (OA; middle), and age differences in this memory effect (bottom). Peak loci of each cluster are reported in the Extended Data (Figure 7-1).

The absence of a trial-wise relationship between distinctiveness and memory could potentially be related to how the regions in which we measured distinctiveness were defined (i.e., based on our clusters showing age differences in distinctiveness). It is possible that this method emphasized the interindividual variability in neural distinctiveness rather than allowing within-person relationships to emerge. Thus, we performed whole-brain searchlight analyses searching for regions in which distinctiveness was greater for subsequently remembered objects than forgotten objects. This analysis yielded no significant clusters with difference in item-level distinctiveness between remembered and forgotten objects in either age group. However, we identified several regions in which category-level distinctiveness demonstrated a subsequent memory effect in both age groups (Fig. 7). In younger adults, we found seven clusters showing this memory effect (*p*s < 0.03), including the bilateral middle occipital, angular, superior parietal, and middle frontal gyri. Older adults showed a subsequent memory effect in four clusters (*p*s < 0.03), including bilateral middle occipital, superior occipital, and angular gyri. Age differences in the subsequent memory effect were observed in two clusters in the right hemisphere (*p*s < 0.05): one in the thalamus and one in the insula and inferior frontal gyrus. In both clusters, younger adults demonstrated a stronger memory effect than older adults.

10.1523/ENEURO.0337-23.2023.f7-1 Figure 7-1 Clusters identified by searchlight similarity analyses revealing greater category-level distinctiveness for subsequently remembered objects than forgotten objects and age differences therein. Download Figure 7-1, DOCX file.

### Neural distinctiveness related across representational levels

**Figure 8.**
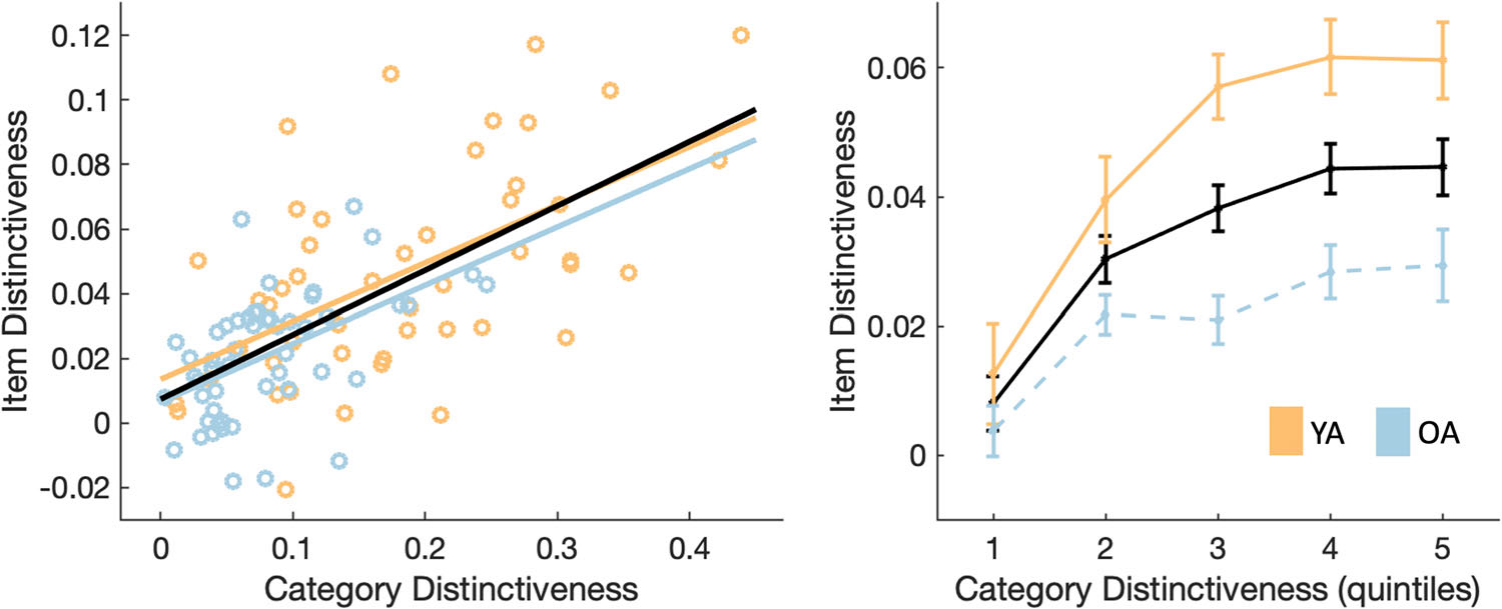
Neural distinctiveness was associated across representational levels both across individuals (left) and within individuals (right). Younger adults (YA) are depicted in orange and older adults (OA) are depicted in blue. Across-group averages are shown in black. Dots represent individual participants in the scatter plot. For visualization, item-level distinctiveness was binned into quintiles according to category-level distinctiveness in each participant. Item-level distinctiveness was then averaged within each age group for each quintile. Error bars indicate ±1 standard error.

In a final step, we examined the relationship between item- and category-level distinctiveness both across and within individuals. Since one of the item-level clusters demonstrated age-related neural dedifferentiation, as did the category-level cluster, we related neural distinctiveness measures extracted from these two clusters. A linear regression model revealed a significant relationship between item- and category-level distinctiveness across individuals (Fig. 8; *R*^2^ = 0.44, *F*_(3, 96)_ = 25.29, *p* < 0.001, adjusted *R*^2^ = 0.42). Note that this relationship remains significant also when standardizing distinctiveness within age group. Category-level distinctiveness (*β* = 0.18, *p* < 0.001) was a significant predictor of item-level distinctiveness. Neither the main effect of age (*β* = −0.01, *p* = 0.41) nor the interaction of age and category-level distinctiveness were significant (*β* = 0.00, *p* = 0.99).

Results of a linear mixed effects model further showed a trial-wise relationship of distinctiveness across representational levels. A main effect of category-level distinctiveness (*t* = 7.33, *p* < 0.001, *d* = 0.88) was identified (Fig. 7). Neither the main effect of age (*t* = −0.90, *p* = 0.37, *d* = 0.02) nor the interaction between age and category-level distinctiveness reached significance (*t* = −0.75, *p* = 0.45, *d* = 0.14). The relationship between item- and category-level distinctiveness remained significant when distinctiveness was standardized within each individual.

## Discussion

While age differences in neural representations on the category level have been reported by several previous studies (Carp et al., 2011; Zheng et al., 2018; Koen et al., 2019; Srokova et al., 2020; Hill et al., 2021; Pauley et al., 2023), evidence for age differences in the specificity of neural representations on the item level is surprisingly meager, despite their potential contribution to age-related memory decline. As a result, there is currently a push to understand how age influences neural representations of individual items (Koen, 2022; Srokova et al., 2023), but evidence so far remains inconclusive. Here, we investigated age differences in neural distinctiveness across category and item representational levels using an fMRI-based paradigm with a subsequent memory test. We found clear evidence not only for age differences in category-, but also in item-level distinctiveness in posterior occipital cortices. Congruent with theories of cognitive aging, neural distinctiveness in regions displaying age-related dedifferentiation was related to interindividual differences in memory performance, and, on the single-trial level, memory performance varied with category-level distinctiveness. Finally, representational distinctiveness across representational levels showed a clear association. In summary, we provide crucial evidence indicating that older adults indeed have less distinct memory representations, also on the item level, which (at least partially) explains older adults’ impairment in remembering specific events. Interestingly, item-level distinctiveness seems to be highly related to category-level distinctiveness, suggestive of a common cause for age-related neural dedifferentiation.

Previous studies have mostly established evidence for neural dedifferentiation at the category level, which indicates that the presentation of stimuli belonging to different categories (here, objects and scenes) evoke more similar neural patterns in older than in younger adults. In contrast, neural dedifferentiation at the item level indicates that neural patterns evoked by different exemplars of the same category are less distinctly represented. Here, we found that younger adults exhibited higher neural distinctiveness than older adults at both the item and category level in early visual cortices, in line with the age-related neural dedifferentiation hypothesis (Li et al., 2001; Koen and Rugg, 2019). The finding of degraded neural representations of highly specific visual information in older adults mirrors behavioral findings showing an age-related reduction in memory precision (Pertzov et al., 2015; Nilakantan et al., 2018; Korkki et al., 2020) and in perceptual discrimination (Ryan et al., 2012; Davidson et al., 2019; Gellersen et al., 2021). Hence, the lack of detail in older adults’ neural representations may result in the inability to discriminate between perceptually similar (yet different) stimuli and loss of detailedness in memories. Furthermore, while some studies have reported age differences in regions selective to a particular stimulus category (e.g., parahippocampal gyrus for scenes), regions typically selective to objects (e.g., lateral occipital cortex) do not seem to show this difference (e.g., Koen et al., 2019). Our findings support these absent age effects in object-selective cortices, and rather suggest that older adults have degraded neural representations in early visual cortices.

Concurrently, we observed higher neural distinctiveness at the item level over posterior parietal cortex in older relative to younger adults. During encoding, occipital cortices have been shown to process detailed perceptual information while parietal cortices process abstracted representations drawn from perceptual experience (for example, semantic knowledge; Jeong and Xu, 2016; Xiao et al., 2017). Thus, our findings suggest that younger adults form more distinct neural representations capturing the perceptual details of an event, whereas older adults form more abstracted (yet still stimulus-specific) representations (see St-Laurent and Buchsbaum, 2019). This interpretation is in line with recent work reporting age-related decreases in distinctiveness (i.e., dedifferentiation) in early visual (perceptual) cortices alongside age-related increases in distinctiveness in higher-order regions associated with semantic memory (Deng et al., 2021; Naspi et al., 2023). The behavioral relevance of this heightened specificity in older age was recently shown by Naspi et al. (2023), who found that highly distinctive semantic representations in older adults supported the perceived vividness of memories. Evidence from behavioral studies has suggested that older adults rely more on semantic memory than episodic memory to support memory performance (e.g., Pitts et al., 2022). For example, Wilson et al. (2018) demonstrated that older adults performed markedly worse on a perceptual interference task than on a semantic interference task, indicating that they were better able to draw on semantic memories than highly-detailed perceptual memories. In concert, these findings point to older adults processing mnemonic information in more abstracted representations rather than highly detailed perceptual representations as with younger adults.

In line with a recent study underscoring the significance of forming highly distinctive neural representations during memory encoding for later successful retrieval (Pauley et al., 2023), higher neural distinctiveness in the calcarine cortex and lingual gyrus was associated with better memory performance across individuals (see also Zheng et al., 2018; Koen et al., 2019; Srokova et al., 2020). This relationship was apparent in both younger and older adults, replicating previous work showing an age-invariant link between distinctiveness and memory (Koen et al., 2019, 2020), indicating that strong neural distinctiveness remains beneficial for memory performance across adulthood. Neural distinctiveness extracted from the regions demonstrating age-related neural dedifferentiation were tied to memory as posited by longstanding theories of cognitive aging (Li et al., 2001), highlighting the role of age-related differences in neural integrity (or degradation) in behavioral deficits. Importantly, both item- and category-level distinctiveness supported memory performance, addressing the otherwise open question as to whether item-level distinctiveness explains interindividual variability in mnemonic ability (Sommer and Sander, 2022). This added link between memory and item-level distinctiveness reveals that individuals who tend to process novel information in highly unique representations are overall less susceptible to memory errors.

We were additionally interested in whether distinctiveness was greater for subsequently remembered compared to forgotten objects. In both age groups, we identified several regions demonstrating subsequent memory effects for category-level distinctiveness, including the bilateral middle occipital, angular, superior parietal, and middle frontal gyri in young adults, and bilateral middle occipital, superior occipital, and angular gyri in older adults, further reinforcing the importance of distinctive neural representations for memory outcomes. In particular, the observed link between distinctiveness in the angular gyrus and mnemonic processing align with recent evidence from Korkki et al. (2023) who showed that both functional and structural measures of the angular gyrus support memory precision during retrieval (see also, Rugg and Vilberg, 2013; Ritchey and Cooper, 2020). While a relationship between intraindividual differences in distinctiveness and memory outcomes has been previously shown in younger adults (Kuhl et al., 2012; Xue et al., 2010, 2013; Lu et al., 2015; Xue, 2018), only a study by Zheng et al. (2018) found evidence for a subsequent memory effect in both younger and older adults. In their study, the authors observed that trial-to-trial variations in item-level distinctiveness predicted memory outcomes. Contrary to this result, we found within-person memory effects for category-level distinctiveness. However, this discrepancy could probably be attributed to a power issue, since we only had two (scanned) presentations of each stimulus in contrast to the three presentations of Zheng et al. (2018). Although subsequent memory effects were present in both age groups (in line with Zheng et al., 2018), we further found that these memory effects were greater in younger compared to older adults in regions including the inferior frontal gyrus. Intriguingly, when taking into account previous reports of a distinctiveness-related memory effect in frontal cortices in younger adults (Kuhl et al., 2012; Xue et al., 2010, 2013), our finding of age differences in this region suggests that reductions in content-specific information of frontal representations may contribute to impaired memory performance in older adults.

Recent studies have demonstrated that categorical distinctions in visual representations may emerge due to overlap in low-level image features across exemplars belonging to a particular category (Long et al., 2018; Konkle and Alvarez, 2022), implying a direct relationship between category- and item-level distinctiveness. As such, unstable item-level representations in older adults may feed forward into reduced distinctiveness at the category level (Sommer and Sander, 2022). However, it is possible that this link between item and category representations is region-specific and limited to rather early visual regions. We observed this phenomenon in posterior occipital cortices, regions often found to represent categories distinctively in whole-brain approaches (Carp et al., 2011; Pauley et al., 2023), but not explicitly considered categorically distinctive in region-of-interest approaches (e.g., fusiform gyrus; Park et al., 2004). Thus, while category-level distinctiveness may manifest as a byproduct of item-level distinctiveness in posterior occipital cortices, higher-level visual cortices (e.g., fusiform and parahippocampal gyri) may have differential representational properties underlying these two levels of distinctiveness. In turn, such a strong relationship between item and category representations may not be uncovered in these categorically-selective cortices. Indeed, recent evidence suggests a null relationship between item- and category-level distinctiveness in the parahippocampal gyrus and lateral occipital cortex (Srokova et al., 2023). More work will be needed both to verify our findings in early visual cortices as well as understand whether and how item- and category-level distinctiveness are interconnected in other neural areas.

Both our inter- and intraindividual analyses bolstered the hypothesis that item- and category-level distinctiveness are similarly compromised during aging, suggesting a tight link between these two measures of dedifferentiation. This finding is also in agreement with the “common cause” explanation of cognitive aging (Lindenberger and Baltes, 1994), which proposes that a single biological mechanism underlies most functional and cognitive decline. Neurobiological modeling revealed deficient dopaminergic modulation as a plausible “common” mechanism (Li and Lindenberger, 1999; Li et al., 2001) associated with age-related memory decline (Bäckman et al., 2006, 2010; Abdulrahman et al., 2017; Rieckmann et al., 2018). Abdulrahman et al. (2017) even connected age differences in neural distinctiveness to an age-related reduction in dopamine (but, for an absent relationship, see Rieckmann et al., 2018). More recently, the contribution of other neurotransmitters to age-related differences has gained interest since researchers observed a link between age differences in neural distinctiveness and reduced gamma-aminobutyric acid (GABA) levels (Lalwani et al., 2019; Chamberlain et al., 2021). Interestingly, some researchers propose that findings of age-related neural dedifferentiation in some regions (e.g., parahippocampal gyrus), but not in others (e.g., fusiform gyrus) challenge the notion that a single mechanism underlies these discrepant effects of age because there is not consistency across neural regions (Koen and Rugg, 2019; Srokova et al., 2020). However, recent evidence showing the wide variation in neurotransmitter concentrations (including dopamine and GABA) across the brain (Hansen et al., 2022) suggests that age-related disruptions in a particular neurotransmitter system could have differential consequences across brain regions. In the present study, we find that similar regions demonstrate age differences in neural distinctiveness for items and categories (the lingual gyrus and calcarine cortex), both of which contribute similarly to age-related memory decline. Thus, from a parsimonious perspective, our findings support the possibility of a common mechanism underlying cognitive decline. Further studies are needed to clarify the neurobiological mechanism(s) underlying age-related neural dedifferentiation and how these relate to manifestations of dedifferentiation across representational levels.

We would like to point out some limitations in the current study. First, as stated in the methods, we dropped some voxels from our analyses due to noisy signal. Thus, we may not have identified all voxels reflecting item- and category-level distinctiveness (and age differences therein). However, since these potential voxels would have demonstrated the same patterns of effects as the identified clusters, we do not believe they would have interfered with the inter- and intraindividual relationships that we reported. Rather, more voxels would have potentially increased the power of our analyses and by dropping them, we may have underestimated some of the reported effects. Second, in order to measure item-level distinctiveness, the same stimulus needs to be presented at least twice in the experiment. Consequently, the perceptual representations during subsequent presentations are potentially conflated with mnemonic signals and it is not possible to disentangle perceptual from mnemonic content. This is, unfortunately, unavoidable in a within-person experimental design, prompting some to investigate age differences in item similarity across individuals (Koen, 2022). Finally, the target-detection task used in the paradigm to keep participants attentive may have confounded findings of neural dedifferentiation (see Koen and Rugg, 2019). Older individuals may have found the task more demanding than younger individuals and accordingly attended the background images to a lesser degree, resulting in less precise neural representations (but, see Sommer et al., 2021). However, systematic investigations into the role of attention in neural distinctiveness and age differences therein are lacking. Although our reported age effects could have potentially been exacerbated due to attentional demands, it is unlikely that attention explains these age differences in neural distinctiveness entirely.

Altogether, our study demonstrates the presence of age-related neural dedifferentiation across representations of individual stimuli as well as categories in early visual brain regions. Crucially, the distinctiveness of categorical information was closely linked with the distinctiveness of individual events, likely indicating a shared neurobiological basis underlying these age effects. Age differences in neural distinctiveness were related to memory performance on both the inter- and intraindividual level, highlighting the importance of forming distinct and stable content-based representations for effective mnemonic processing. In sum, the findings support the hypothesis of overall reductions in the quality of neural representations as a potential neural basis leading to reduced distinctiveness for both items and categories and subsequent cognitive decline in older age.

## Synthesis

Reviewing Editor: Alexander Soutschek, Ludwig-Maximilians-Universitat Munchen Decisions are customarily a result of the Reviewing Editor and the peer reviewers coming together and discussing their recommendations until a consensus is reached. When revisions are invited, a fact-based synthesis statement explaining their decision and outlining what is needed to prepare a revision will be listed below. The following reviewer(s) agreed to reveal their identity: Paul Hill, Helena Gellersen.

Reviewer #1

The authors report findings from an fMRI study examining age-related neural dedifferentiation at the category- and item-levels of analysis. Age differences in category-level neural distinctiveness were evident in a large occipital cluster (young > older). A similar age difference was identified at the item level in a highly overlapping (albeit spatially smaller) cluster. Item-level distinctiveness was greater in older compared to younger adults in the posterior parietal cortex. The authors report a between-subjects correlation between neural distinctiveness in these regions and memory performance. Likewise, item- and category-level measures of neural distinctiveness were correlated with one another, both between subjects and within-subjects at the trialwise level. The paper is timely, and the authors do a good job of describing the paucity of studies examining neural dedifferentiation at the level of individual items.

1. There are two recent papers examining item-level neural dedifferentiation that the authors should consider citing. I realize the second paper is a preprint, but certainly worth considering given the similar scope of this submission. You might also consider citing some of the work from Nancy Dennis' lab when discussing category-level dedifferentiation.

• Koen J. (2022). Age-related neural dedifferentiation for individual stimuli: an across participant pattern similarity analysis. Aging, Neuropsychology, and Cognition

• Srokova et al. (2023). Dissociative effects of age on neural differentiation at the category and item level. bioRxiv.

2. Memory performance was calculated as the proportion of correct item (recognition) or object-scene pair (associative memory) responses. It would be helpful to also report the false alarm rate, as participant could simply endorse each item as 'old' to achieve a high hit rate.

3. It might be helpful to report the peak loci of the clusters identified by the search light analyses.

4. For the target detection task, participants were required to respond if/when the fixation cross turned red. In theory, participants can simply ignore the background image and focus attention exclusively on the cross. This introduces a potential age-related attentional confound into the task. On a similar note, each block consisted of images from the same stimulus class (all objects or all scenes). Using this kind of blocked design raises the possibility that some form of attentional orientation towards a specific visual category may have produced sustained activity in object- or scene-selective cortex (this would seemingly be more of an issue for computing category distinctiveness rather than item-level).

5. When computing distinctiveness at the item level, how can the authors rule out the possibility of a recognition or familiarity-related signal at the second presentation? Whereas the first presentation might be highly perceptual, the second presentation might now be conflated with mnemonic signals. This is admittedly one of the reasons item-level distinctiveness is difficult to isolate.

6. As someone not readily familiar with partial least squares analysis, I'm curious whether the correlations between neural distinctiveness and memory performance are age-dependent or age-independent? This would be helpful to know, as several prior studies have shown that the relationship between category-level neural dedifferentiation and memory performance is age invariant. Similarly, is there any way to confirm which of the three identified brain regions are driving this effect? What is the rationale for running this analysis across all three brain regions, rather than separately for the respective clusters? Am I correct in assuming the BSRs plotted in the middle panel of Figure 6 confirm that the item- and category-level clusters identified in young adults (which were largely overlapping in the occipital cortex) are driving the significant LV? The scatter plots presented in the right panel of Figure 6 also seem misleading, as you have large age differences in both the latent memory scores and the latent distinctiveness scores. Would it be more accurate to plot the residuals of these scores after controlling for age?

7. The PLS and various linear mixed effects analyses often combine data from across different brain regions. Although you do mention this in the methods, it was very hard to follow when reading through the results and I found myself going back and forth as I was going through the paper. I'm also left wondering about the rationale of combining across ROIs. What happens when you run these separately?

8. First line of the Discussion the authors note that category-level dedifferentiation has been reported by numerous prior studies - might be worth citing those studies here.

9. I'm not sure I follow how the relationship between item- and category-level neural dedifferentiation offers strong support for the common cause hypothesis of cognitive aging. Why does it necessarily follow that the two levels of representation are dependent on a common neurobiological mechanism? Could it not be just as likely that the relationship between these measures is simply capturing some shared source of voxel-wise noise?

Reviewer #2

The authors fill an important gap in the literature on memory and neural distinctiveness in ageing, namely that of item-level representational distinctiveness and its effect on memory performance. They use an intelligent experimental design to disentangle the effects of category- and item-level neural distinctiveness on memory. Their findings provide further insight into mechanisms behind memory deficits in ageing that can explain the common findings of reduced perceptual and mnemonic fidelity in the face of preserved gist-based memory recall.

1) The spatially specific signal loss described on page 9 suggests that in some regions it will have been more difficult, if possible at all, to detect neural distinctiveness effects. The parahippocampal gyrus (in particular perirhinal, parahippocampal cortex) comes to mind given that the strong category-selectivity of its subregions (e.g. Barense et al., 2009 Hippocampus) positions them well to show the expected category distinctiveness effects reported in the present study. It would be important to provide more specific information with respect to the regions that suffered from particularly poor signal and note this as a limitation in the discussion.

2) Please add effect sizes to the statistical analyses, e.g. in the ANOVA results, partial eta squared should be indicated for the model effects. Pairwise comparisons can be similarly supplemented with effect sizes such as Cohen's d (e.g. see R packages such as effectsize). For pairwise comparisons, estimated marginal means from the model and their CIs should also be included.

3) Page 18: "Younger adults demonstrated modest correlations for both item and pair memory (r = -0.46 and -0.38, respectively), while older adults had weak correlations for item and pair memory (r = -0.33 and -0.04, respectively)." Please state explicitly that these correlations refer to the LV (from context, I presume that this is what is meant but I might be wrong. Hence, clarification would be good).

4) Figure 6: please show confidence intervals around the linear trend lines.

5) With respect to the author's interpretation of their findings on page 22 ("During encoding, occipital cortices have been shown to process detailed perceptual information while parietal cortices process abstracted representations drawn from perceptual experience (for example, semantic knowledge; Jeong & Xu, 2016; Xiao et al., 2017). Thus, our findings suggest that younger adults form more distinct neural representations capturing the perceptual details of an event, whereas older adults form more abstracted (yet still stimulus-specific) representations (see St-Laurent & Buchsbaum, 2019).": see a recent study by Naspi et al. (2023, Journal of Neuroscience) who looked at visual vs. semantic representations in ageing and their relationship with memory vividness (although not based on parietal cortical activation but temporal cortex). Moreover, Korkki et al. (2023, Neurobiol Aging) recently showed age-related reductions in memory retrieval precision effects in the angular gyrus which are relevant when considering category-level distinctiveness effects observed in YAs as shown in Figure 7 top panel given that these regions seem to overlap.

6) Seeing the authors concluding that "older adults process mnemonic information in more abstracted representations rather than highly detailed perceptual representations as with younger adults.", it would be good to reference at least briefly the broad behavioural literature on age-related changes in perceptual and memory fidelity and how the current findings can inform this literature. For example, Wilson et al. (2018 Neuropsychologia) show that perceptual, not semantic interference negatively impacts memory in older adults which would be in line with the authors' assertion that older adults' memory representations are more abstract and conceptual than perceptually detailed. Moreover, age-related reductions in the distinctiveness of item-level representations found by the authors of the present study could explain findings of reduced memory precision in older adults both in short- and long-term memory (as shown before in multiple studies such as Gellersen et al, 2023 psyrxiv; Korkki et al. 2020 Psych & Aging; Nilakantan et al. 2018 Neuropsychologia; Pertzov et al., 2015 Psych & Aging; Rhodes et al., 2020 Psych & Aging), but also deficits in perceptual representational quality and its relationship with memory performance (Davidson et al. 2019 J Geront Series B; Gellersen et al., 2021 Cognition; Ryan et al., 2012 Hippocampus). This could, for example, also explain why older adults did not perform worse than younger adults on the object identification task tested here in the present study given that objects were not perceptually similar and therefore less detailed representations would be sufficient to support adequate performance (e.g. Stark et al., 2015 Behavioural Neuroscience).

## References

[B1] Abdulrahman H, Fletcher PC, Bullmore E, Morcom AM (2017) Dopamine and memory dedifferentiation in aging. NeuroImage 153:211–220. 10.1016/j.neuroimage.2015.03.031 25800211 PMC5460975

[B2] Bäckman L, Lindenberger U, Li SC, Nyberg L (2010) Linking cognitive aging to alterations in dopamine neurotransmitter functioning: recent data and future avenues. Neurosci Biobehav Rev 34:670–677. 10.1016/j.neubiorev.2009.12.008 20026186

[B3] Bäckman L, Nyberg L, Lindenberger U, Li SC, Farde L (2006) The correlative triad among aging, dopamine, and cognition: current status and future prospects. Neurosci Biobehav Rev 30:791–807. 10.1016/j.neubiorev.2006.06.005 16901542

[B4] Bowman CR, Chamberlain JD, Dennis NA (2019) Sensory representations supporting memory specificity: age effects on behavioral and neural discriminability. J Neurosci 39:2265–2275. 10.1523/JNEUROSCI.2022-18.2019 30655350 PMC6433767

[B5] Brady TF, Konkle T, Alvarez GA, Oliva A (2008) Visual long-term memory has a massive storage capacity for object details. Proc Natl Acad Sci U S A 105:14325–14329. 10.1073/pnas.0803390105 18787113 PMC2533687

[B6] Carp J, Park J, Polk TA, Park DC (2011) Age differences in neural distinctiveness revealed by multi-voxel pattern analysis. NeuroImage 56:736–743. 10.1016/j.neuroimage.2010.04.267 20451629 PMC2962693

[B7] Chamberlain JD, Gagnon H, Lalwani P, Cassady KE, Simmonite M, Seidler RD, Taylor SF, Weissman DH, Park DC, Polk TA (2021) GABA levels in ventral visual cortex decline with age and are associated with neural distinctiveness. Neurobiol Aging 102:170–177. 10.1016/j.neurobiolaging.2021.02.013 33770531 PMC8205971

[B8] Davidson PSR, Vidjen P, Trincao-Batra S, Collin CA (2019) Older adults’ lure discrimination difficulties on the mnemonic similarity task are significantly correlated with their visual perception. J Gerontol Ser B 74:1298–1307. 10.1093/geronb/gby130 30407604

[B9] Deng L, Davis SW, Monge ZA, Wing EA, Geib BR, Raghunandan A, Cabeza R (2021) Age-related dedifferentiation and hyperdifferentiation of perceptual and mnemonic representations. Neurobiol Aging 106:55–67. 10.1016/j.neurobiolaging.2021.05.021 34246857 PMC12268951

[B10] Dennis N, Koen J (2022) Introduction to the special issue: advances in understanding the cognitive neuroscience of aging with multivariate methods. Aging Neuropsychol Cogn 29:367–374. 10.1080/13825585.2022.2044447 35343386

[B11] Dimsdale-Zucker HR, Ranganath C (2018) Representational similarity analyses: a practical guide for functional MRI applications. Handb Behav Neurosci 28:509–525. 10.1016/B978-0-12-812028-6.00027-6

[B12] Esteban O, et al. (2019) fMRIPrep: a robust preprocessing pipeline for functional MRI. Nat Methods 16:111–116. 10.1038/s41592-018-0235-4 30532080 PMC6319393

[B13] Folstein MF, Folstein SE, McHugh PR (1975) “Mini-mental state”. A practical method for grading the cognitive state of patients for the clinician. J Psychiatr Res 12:189–198. 10.1016/0022-3956(75)90026-6 1202204

[B14] Gellersen HM, Trelle AN, Henson RN, Simons JS (2021) Executive function and high ambiguity perceptual discrimination contribute to individual differences in mnemonic discrimination in older adults. Cognition 209:104556. 10.1016/j.cognition.2020.104556 33450438 PMC8223497

[B15] Gorgolewski KJ, et al. (2016) The brain imaging data structure, a format for organizing and describing outputs of neuroimaging experiments. Sci Data 3:160044. 10.1038/sdata.2016.44 27326542 PMC4978148

[B16] Gregorich M, Strohmaier S, Dunkler D, Heinze G (2021) Regression with highly correlated predictors: variable omission is not the solution. Int J Environ Res Public Health 18.4259. 10.3390/ijerph18084259 33920501 PMC8073086

[B17] Hansen JY, et al. (2022) Mapping neurotransmitter systems to the structural and functional organization of the human neocortex. Nat Neurosci 25:1569–1581. 10.1038/s41593-022-01186-3 36303070 PMC9630096

[B18] Hasinski AE, Sederberg PB (2016) Trial-level information for individual faces in the fusiform face area depends on subsequent memory. NeuroImage 124:526–535. 10.1016/j.neuroimage.2015.08.065 26343317

[B19] Hill PF, King DR, Rugg MD (2021) Age differences in retrieval-related reinstatement reflect age-related dedifferentiation at encoding. Cereb Cortex 31:106–122. 10.1093/cercor/bhaa210 32829396 PMC7727391

[B20] Jeong SK, Xu Y (2016) Behaviorally relevant abstract object identity representation in the human parietal cortex. J Neurosci 36:1607–1619. 10.1523/JNEUROSCI.1016-15.2016 26843642 PMC4737772

[B21] Karlsson AE, Lindenberger U, Sander MC (2022) Out of rhythm: compromised precision of theta-gamma coupling impairs associative memory in old age. J Neurosci 42:1752–1764. 10.1523/JNEUROSCI.1678-21.2021 34996815 PMC8896557

[B22] Karlsson AE, Sander MC (2023) Altered alpha/beta desynchronization during item–context binding contributes to the associative deficit in older age. Cereb Cortex 33:2455–2469. 10.1093/cercor/bhac219 35750026 PMC10016059

[B23] Koen JD (2022) Age-related neural dedifferentiation for individual stimuli: an across-participant pattern similarity analysis. Aging Neuropsychol Cogn 29:552–576. 10.1080/13825585.2022.2040411 PMC8960356 35189773

[B24] Koen JD, Hauck N, Rugg MD (2019) The relationship between age, neural differentiation, and memory performance. J Neurosci 39:149–162. 10.1523/JNEUROSCI.1498-18.2018 30389841 PMC6325265

[B25] Koen JD, Rugg MD (2019) Neural dedifferentiation in the aging brain. Trends Cogn Sci 23:547–559. 10.1016/j.tics.2019.04.012 31174975 PMC6635135

[B26] Koen JD, Srokova S, Rugg MD (2020) Age-related neural dedifferentiation and cognition. Curr Opin Behav Sci 32:7–14. 10.1016/j.cobeha.2020.01.006 32095492 PMC7039299

[B27] Konkle T, Alvarez GA (2022) A self-supervised domain-general learning framework for human ventral stream representation. Nat Commun 13:491. 10.1038/s41467-022-28091-4 35078981 PMC8789817

[B29] Korkki SM, Richter FR, Jeyarathnarajah P, Simons JS (2020) Healthy ageing reduces the precision of episodic memory retrieval. Psychol Aging 35:124–142. 10.1037/pag0000432 31928030

[B28] Korkki SM, Richter FR, Gellersen HM, Simons JS (2023) Reduced memory precision in older age is associated with functional and structural differences in the angular gyrus. Neurobiol Aging 129:109–120. 10.1016/j.neurobiolaging.2023.04.009 37300913

[B30] Krishnan A, Williams LJ, McIntosh AR, Abdi H (2011) Partial least squares (PLS) methods for neuroimaging: a tutorial and review. NeuroImage 56:455–475. 10.1016/j.neuroimage.2010.07.034 20656037

[B31] Kuhl BA, Rissman J, Wagner AD (2012) Multi-voxel patterns of visual category representation during episodic encoding are predictive of subsequent memory. Neuropsychologia 50:458. 10.1016/j.neuropsychologia.2011.09.002 21925190 PMC3357999

[B32] Lalwani P, Gagnon H, Cassady K, Simmonite M, Peltier S, Seidler RD, Taylor SF, Weissman DH, Polk TA (2019) Neural distinctiveness declines with age in auditory cortex and is associated with auditory GABA levels. NeuroImage 201:116033. 10.1016/j.neuroimage.2019.116033 31326572 PMC6765441

[B33] Li S-C, Lindenberger U (1999) Cross-level unification: a computational exploration of the link between deterioration of neurotransmitter systems and dedifferentiation of cognitive abilities in old age. In: Cognitive neuroscience of memory (Nilsson L-G, Markowitsch HJ, eds), pp 103–146.

[B34] Li S-C, Lindenberger U, Sikström S (2001) Aging cognition: from neuromodulation to representation. Trends Cogn Sci 5:479–486. 10.1016/S1364-6613(00)01769-1 11684480

[B35] Lindenberger U, Baltes PB (1994) Sensory functioning and intelligence in old age: a strong connection. Psychol Aging 9:339–355. 10.1037/0882-7974.9.3.339 7999320

[B36] Long B, Yu C-P, Konkle T (2018) Mid-level visual features underlie the high-level categorical organization of the ventral stream. Proc Natl Acad Sci U S A 115:E9015–E9024. 10.1073/pnas.1719616115 30171168 PMC6156638

[B37] Lu Y, Wang C, Chen C, Xue G (2015) Spatiotemporal neural pattern similarity supports episodic memory. Curr Biol 25:780–785. 10.1016/j.cub.2015.01.055 25728695

[B38] Maass A, et al. (2019) Alzheimer’s pathology targets distinct memory networks in the ageing brain. Brain J Neurol 142:2492–2509. 10.1093/brain/awz154 PMC6658874 31199481

[B39] Naspi L, Stensholt C, Karlsson AE, Monge ZA, Cabeza R (2023) Effects of aging on successful object encoding: enhanced semantic representations compensate for impaired visual representations. J Neurosci. 43:7337–7350. 10.1523/JNEUROSCI.2265-22.2023 37673674 PMC10621770

[B40] Nilakantan AS, Bridge DJ, VanHaerents S, Voss JL (2018) Distinguishing the precision of spatial recollection from its success: evidence from healthy aging and unilateral mesial temporal lobe resection. Neuropsychologia 119:101. 10.1016/j.neuropsychologia.2018.07.035 30086364 PMC6191347

[B41] Nili H, Wingfield C, Walther A, Su L, Marslen-Wilson W, Kriegeskorte N (2014) A toolbox for representational similarity analysis. PLOS Comput Biol 10:e1003553. 10.1371/journal.pcbi.1003553 24743308 PMC3990488

[B42] Oostenveld R, Fries P, Maris E, Schoffelen JM (2011) FieldTrip: open source software for advanced analysis of MEG, EEG, and invasive electrophysiological data. Comput Intell Neurosci 2011:156869. 10.1155/2011/156869 21253357 PMC3021840

[B43] Park DC, Polk TA, Park R, Minear M, Savage A, Smith MR (2004) Aging reduces neural specialization in ventral visual cortex. Proc Natl Acad Sci U S A 101:13091–13095. 10.1073/pnas.0405148101 15322270 PMC516469

[B44] Pauley C, Kobelt M, Werkle-Bergner M, Sander MC (2023) Age differences in neural distinctiveness during memory encoding, retrieval, and reinstatement. Cereb Cortex 33:9489–9503. 10.1093/cercor/bhad219 37365853 PMC10431749

[B45] Pertzov Y, Heider M, Liang Y, Husain M (2015) Effects of healthy ageing on precision and binding of object location in visual short term memory. Psychol Aging 30:26–35. 10.1037/a0038396 25528066 PMC4360752

[B46] Pitts BL, Smith ME, Newberry KM, Bailey HR (2022) Semantic knowledge attenuates age-related differences in event segmentation and episodic memory. Mem Cognit 50:586–600. 10.3758/s13421-021-01220-y PMC8938297 34553341

[B47] Power JD (2017) A simple but useful way to assess fMRI scan qualities. NeuroImage 154:150–158. 10.1016/j.neuroimage.2016.08.009 27510328 PMC5296400

[B48] Rieckmann A, Johnson KA, Sperling RA, Buckner RL, Hedden T (2018) Dedifferentiation of caudate functional connectivity and striatal dopamine transporter density predict memory change in normal aging. Proc Natl Acad Sci U S A 115:10160–10165. 10.1073/pnas.1804641115 30224467 PMC6176586

[B49] Ritchey M, Cooper RA (2020) Deconstructing the posterior medial episodic network. Trends Cogn Sci 24:451–465. 10.1016/j.tics.2020.03.006 32340798

[B50] Rugg MD, Vilberg KL (2013) Brain networks underlying episodic memory retrieval. Curr Opin Neurobiol 23:255–260. 10.1016/j.conb.2012.11.005 23206590 PMC3594562

[B51] Ryan L, Cardoza JA, Barense MD, Kawa KH, Wallentin-Flores J, Arnold WT, Alexander GE (2012) Age-related impairment in a complex object discrimination task that engages perirhinal cortex. Hippocampus 22:1978–1989. 10.1002/hipo.22069 22987676 PMC4512648

[B52] Snodgrass JG, Corwin J (1988) Pragmatics of measuring recognition memory: applications to dementia and amnesia. J Exp Psychol Gen 117:34–50. 10.1037/0096-3445.117.1.34 2966230

[B54] Sommer VR, Sander MC (2022) Contributions of representational distinctiveness and stability to memory performance and age differences. Aging Neuropsychol Cogn 29:443–462. 10.1080/13825585.2021.2019184 34939904

[B53] Sommer VR, Mount L, Weigelt S, Werkle-Bergner M, Sander MC (2021) Memory specificity is linked to repetition effects in event-related potentials across the lifespan. Dev Cogn Neurosci 48:100926. 10.1016/j.dcn.2021.100926 33556880 PMC7868631

[B56] Srokova S, Hill PF, Koen JD, King DR, Rugg MD (2020) Neural differentiation is moderated by age in scene-selective, but not face-selective, cortical regions. eNeuro 7:ENEURO.0142-20.2020. 10.1523/ENEURO.0142-20.2020 PMC7242814 32341120

[B55] Srokova S, Aktas ANZ, Koen JD, Rugg MD (2023) Dissociative effects of age on neural differentiation at the category and item level. J Neurosci 44:e0959232023. 10.1523/JNEUROSCI.0959-23.2023 PMC10860568 38050137

[B57] St-Laurent M, Buchsbaum BR (2019) How multiple retrievals affect neural reactivation in young and older adults. J Gerontol B Psychol Sci Soc Sci 74:1086–1100. 10.1093/geronb/gbz075 31155678 PMC6748716

[B58] Trelle AN, Henson RN, Simons JS (2019) Neural evidence for age-related differences in representational quality and strategic retrieval processes. Neurobiol Aging 84:50–60. 10.1016/j.neurobiolaging.2019.07.012 31491595 PMC6805220

[B59] Tzourio-Mazoyer N, Landeau B, Papathanassiou D, Crivello F, Etard O, Delcroix N, Mazoyer B, Joliot M (2002) Automated anatomical labeling of activations in SPM using a macroscopic anatomical parcellation of the MNI MRI single-subject brain. NeuroImage 15:273–289. 10.1006/nimg.2001.0978 11771995

[B60] Wilson DM, Potter KW, Cowell RA (2018) Recognition memory shielded from semantic but not perceptual interference in normal aging. Neuropsychologia 119:448–463. 10.1016/j.neuropsychologia.2018.07.031 30071206

[B61] Xiao X, Dong Q, Gao J, Men W, Poldrack RA, Xue G (2017) Transformed neural pattern reinstatement during episodic memory retrieval. J Neurosci 37:2986–2998. 10.1523/JNEUROSCI.2324-16.2017 28202612 PMC6596730

[B62] Xue G (2018) The neural representations underlying human episodic memory. Trends Cogn Sci 22:544–561. 10.1016/j.tics.2018.03.004 29625850

[B63] Xue G, Dong Q, Chen C, Lu Z, Mumford JA, Poldrack RA (2010) Greater neural pattern similarity across repetitions is associated with better memory. Science 330:97–101. 10.1126/science.1193125 20829453 PMC2952039

[B64] Xue G, Dong Q, Chen C, Lu Z-L, Mumford JA, Poldrack RA (2013) Complementary role of frontoparietal activity and cortical pattern similarity in successful episodic memory encoding. Cereb Cortex 23:1562–1571. 10.1093/cercor/bhs143 22645250 PMC3726068

[B65] Zheng L, Gao Z, Xiao X, Ye Z, Chen C, Xue G (2018) Reduced fidelity of neural representation underlies episodic memory decline in normal aging. Cereb Cortex 28:2283–2296. 10.1093/cercor/bhx130 28591851

