# Supplementary material for: Early Visual Cortices Reveal Interrelated Item and Category Representations in Aging
Source: eNeuro. 2024 Mar 12;11(3):ENEURO.0337-23.2023. doi: 10.1523/ENEURO.0337-23.2023 (PMC10960632; doi:10.1523/ENEURO.0337-23.2023)
Supplement: Figure 4-1 — Clusters identified by searchlight similarity analyses revealing high item- and category-level distinctiveness in younger and older adults. Download Figure 4-1, DOCX file. [file eneuro-11-ENEURO.0337-23.2023-s003.docx]

Figure 4-1. Clusters identified by searchlight similarity analyses revealing high item- and category-level distinctiveness in younger and older adults.

|  |  |  | **Peak MNI** | | |  |
| --- | --- | --- | --- | --- | --- | --- |
| **Searchlight** | **Regions (AAL)** | **H** | **X** | **Y** | **Z** | **Peak *t*** |
| **Item-level**  ***(Younger adults)*** | Middle occipital gyrus, fusiform gyrus, lingual gyrus | B | 17 | -97 | 15 | 11.47 |
| **Item-level**  ***(Older adults)*** | Middle occipital gyrus, fusiform gyrus, lingual gyrus, superior occipital gyrus | B | -15 | -84 | -10 | 12.66 |
|  | Precentral gyrus, inferior frontal gyrus, middle frontal gyrus | R | 47 | 9 | 22 | 6.29 |
|  | Inferior frontal gyrus, middle frontal gyrus, insula | R | 46 | 21 | 0 | 5.40 |
|  | Inferior frontal gyrus, precentral gyrus, middle frontal gyrus | L | -41 | -4 | 34 | 5.28 |
| **Category-level** | Middle occipital gyrus, lingual gyrus, fusiform gyrus | B | 25 | -83 | -7 | 11.84 |
| ***(Younger adults)*** | Precentral gyrus, inferior frontal gyrus, middle frontal gyrus | L | -48 | 7 | 31 | 6.33 |
|  | Precentral gyrus, inferior frontal gyrus, middle frontal gyrus | R | 40 | 10 | 32 | 5.88 |
|  | Middle temporal gyrus, superior temporal gyrus | L | -53 | -36 | 0 | 4.93 |
|  | Inferior frontal gyrus, middle frontal gyrus | R | 31 | 36 | -10 | 5.15 |
|  | Supramarginal gyrus, postcentral gyrus | R | 47 | -29 | 35 | 4.52 |
|  | Inferior frontal gyrus, middle frontal gyrus | L | -33 | 39 | -12 | 4.77 |
| **Category-level** | Middle occipital gyrus, lingual gyrus, fusiform gyrus | B | -9 | -80 | -9 | 11.21 |
| ***(Older adults)*** | Precentral gyrus, inferior frontal gyrus, middle frontal gyrus, superior frontal gyrus | L | -42 | 2 | 34 | 7.25 |
|  | Inferior frontal gyrus, precentral gyrus, middle frontal gyrus | R | 34 | 43 | 10 | 5.94 |
|  | Inferior frontal gyrus, middle frontal gyrus, insula, superior frontal gyrus | L | -29 | 35 | -11 | 5.45 |
