# Supplementary material for: Early Visual Cortices Reveal Interrelated Item and Category Representations in Aging
Source: eNeuro. 2024 Mar 12;11(3):ENEURO.0337-23.2023. doi: 10.1523/ENEURO.0337-23.2023 (PMC10960632; doi:10.1523/ENEURO.0337-23.2023)
Supplement: Figure 5-1 — Clusters revealed by searchlight similarity analyses demonstrating age differences in item- and category-level distinctiveness. Download Figure 5-1, DOCX file. [file eneuro-11-ENEURO.0337-23.2023-s004.docx]

Figure 5-1. Clusters revealed by searchlight similarity analyses demonstrating age differences in item- and category-level distinctiveness.

|  |  |  | **Peak MNI** | | |  |
| --- | --- | --- | --- | --- | --- | --- |
| **Searchlight** | **Regions** | **H** | **X** | **Y** | **Z** | **Peak *t*** |
| **Item-level** | Angular gyrus, inferior parietal cortex, superior parietal cortex | R | 29 | -68 | 54 | -4.72 |
|  | Calcarine cortex, lingual gyrus | B | 2 | -86 | -6 | 5.98 |
| **Category-level** | Lingual gyrus, calcarine cortex | B | -12 | -77 | 0 | 7.14 |
