# Supplementary material for: Early Visual Cortices Reveal Interrelated Item and Category Representations in Aging
Source: eNeuro. 2024 Mar 12;11(3):ENEURO.0337-23.2023. doi: 10.1523/ENEURO.0337-23.2023 (PMC10960632; doi:10.1523/ENEURO.0337-23.2023)
Supplement: Figure 7-1 — Clusters identified by searchlight similarity analyses revealing greater category-level distinctiveness for subsequently remembered objects than forgotten objects and age differences therein. Download Figure 7-1, DOCX file. [file eneuro-11-ENEURO.0337-23.2023-s002.docx]

Figure 7-1. Clusters identified by searchlight similarity analyses revealing greater category-level distinctiveness for subsequently remembered objects than forgotten objects and age differences therein.

|  |  |  | **Peak MNI** | | |  |
| --- | --- | --- | --- | --- | --- | --- |
| **Searchlight** | **Regions (AAL)** | **H** | **X** | **Y** | **Z** | **Peak *t*** |
| **Younger adults** | Middle occipital gyrus, inferior parietal gyrus, superior parietal gyrus, inferior occipital gyrus fusiform gyrus, lingual gyrus | L | -37 | -47 | 45 | 5.98 |
|  | Superior parietal gyrus, inferior parietal gyrus, angular gyrus | R | 31 | -59 | 59 | 5.51 |
|  | Precentral gyrus, inferior frontal gyrus | L | -48 | 3 | 32 | 5.80 |
|  | Middle occipital gyrus, superior occipital gyrus | R | 29 | -74 | 17 | 5.75 |
|  | Fusiform gyrus, lingual gyrus, inferior occipital gyrus | R | 25 | -59 | -11 | 5.69 |
|  | Precentral gyrus, inferior frontal gyrus | R | 43 | 2 | 32 | 5.22 |
|  | Middle frontal gyrus, inferior frontal gyrus | R | 38 | 39 | 10 | 4.40 |
| **Older adults** | Middle occipital gyrus, superior occipital gyrus | R | 30 | -73 | 28 | 4.41 |
|  | Angular gyrus, middle occipital gyrus, superior occipital gyrus | L | -49 | -72 | 27 | 5.34 |
|  | Inferior occipital gyrus, fusiform gyrus, inferior temporal gyrus, middle occipital gyrus, middle temporal gyrus | L | -35 | -72 | -11 | 5.26 |
|  | Superior parietal gyrus, angular gyrus, inferior parietal gyrus | R | 27 | -70 | 60 | 4.87 |
| **Age differences** | Thalamus | R | 1 | -32 | 17 | 5.09 |
|  | Insula, inferior frontal gyrus | R | 39 | 19 | 6 | 4.42 |
